# Analysis of Larch-Bark Capacity for Formaldehyde Removal in Wood Adhesives

**DOI:** 10.3390/ijerph17030764

**Published:** 2020-01-25

**Authors:** Eugenia Mariana Tudor, Marius Catalin Barbu, Alexander Petutschnigg, Roman Réh, Ľuboš Krišťák

**Affiliations:** 1 Forest Products Technology and Timber Construction Department, Salzburg University of Applied Sciences, Markt 136a, 5431 Kuchl, Austria; eugenia.tudor@fh-salzburg.ac.at (E.M.T.); marius.barbu@fh-salzburg.ac.at (M.C.B.); alexander.petutschnigg@fh-salzburg.ac.at (A.P.); 2 Faculty of Wood Engineering, Transilvania University of Brasov, Bld. Eroilor nr.29, 500036 Brasov, Romania; 3 Faculty of Wood Sciences and Technology, Technical University in Zvolen, T. G. Masaryka 24, SK-960 01 Zvolen, Slovak Republic; reh@tuzvo.sk

**Keywords:** tree bark, decorative panels, formaldehyde content, super E0, perforator method

## Abstract

Ecofriendly wood-based materials are required by consumers at present. Decorative panels are part of a large group of wood-composite materials, and their environmental properties must not be neglected. More environmentally friendly decorative panels can be achieved by various methods. This paper describes a method of production from larch bark. Tree bark, as a byproduct of the wood industry, is one of the research topics that have gained interest in the last decade, especially for its applications in biobased lignocomposites, with regard to the shrinkage of wood resources. In the present work, the formaldehyde content of decorative boards based on larch bark (0.6 g/cm^3^) was analyzed when bonded with five different types of adhesive systems: urea-formaldehyde, polyvinyl acetate, the mixture of 70% urea-formaldehyde + 30% polyvinyl acetate, polyurethane, and tannin-based adhesive. A self-agglomerated board was also analyzed. The formaldehyde content of the larch-bark samples was determined with the perforator method (EN 120:2011), and findings showed that all tested samples reached the E1 classification (≤8 mg/100 oven dry). Moreover, 75% of the values of the corrected formaldehyde content were included in the super-E0 class (≤1.5 mg/100 oven dry). In the case of boards bonded with tannin-based adhesive, this natural polymer acted as a formaldehyde scavenger.

## 1. Introduction

In the last decade, approaches regarding healthy environments and welfare have had increasing attention from both society and the authorities, pointing out that quality of life is also reflected in the building sector [1]. Recent urban development entails safeguarding ecological integrity with the low carbon footprints of construction materials [2]. Nowadays, both employed and unemployed persons spend about 75% of their existence indoors, e.g., at home, in workplaces, schools, and other institutions [3], and sick building syndrome (SBS) has become a severe global problem [1,4,5]. Regarding the main causes of SBS, one can include volatile organic compounds (e.g., formaldehyde) discharged from the adhesives, finishing materials, paint used for furniture and buildings, and building materials (e.g., insulation) [5]. Many materials can absorb formaldehyde released into the atmosphere, e.g., wool [6], ornamental plants [7,8], building materials with adsorption potentials [9], or tree bark [5,10]. 

On a global scale, over 50% of the bark amount resulting from industrially processed logs serves for energy generation [11]. The bark percentage varies depending on tree species, age, and diameter [12]. Analysis by Mikolajczak [13] revealed that, from each 100 m^3^ of harvested tree parts (including twigs, branches, and fresh stumps), 10 m^3^ represents the bark: 50% of the bark is generated from the production of wood fibers (especially for the pulp and paper industry), 23% results after debarking in sawmills, and the same amount is reached after round wood debarking for the production of wood-based composites.

Various studies were carried out to analyze formaldehyde absorption by bark in the cases of particleboard (PB) [14,15,16], plywood [17,18,19], medium-density fiberboard (MDF) [20], and insulation panels using bark and bark extractives [21]. In this study, the bark of the European larch (*Larix decidua* Mill.) was used to produce decorative panels with reduced levels of formaldehyde emissions and content. This larch species was chosen due to general high-scale production in the Alps area, especially in the Federal State of Salzburg, and in order to pay more attention to an underutilized material [11] with thermal-insulation properties [21,22]. Larch is well-represented in other Central European countries. Other reasons for choosing larch bark for research were that larch has a faster growth rate than other conifer species [23], and it produces more wood and bark in less time than other softwoods (which grow faster than hardwoods). The chemical composition of larch bark as compared to that of other softwoods provided the prerequisites for its successful application; the first tests confirmed this assumption. Larch bark contains various extractives. The following hot-water-soluble extracts were obtained: 35% sugar, especially glucose [24]; 30% lignin; 10% to 12% tannic acids and about 4% methoxyl [25]; 3% resin; and 13% arabino-galactan, lignans, resins, and volatile oil (mainly alpha- and betapinene and limonene) [26]. Tannins from the larch bark can be used as a cost-effective substitute for phenol in particleboard adhesives [27]. Such tannin glue that has successfully passed outdoor aging tests may also be suitable for industrial use [28]. Compared to wood, bark is rather more heterogeneous in terms of the proportion and composition of the main components.

Buyuksari [26] studied the influence of particleboard (PB) bonded with urea-formaldehyde (UF) on formaldehyde content when pine-cone flour was added, with values of formaldehyde content (FC) that ranged from 1.99 to 2.48 mg/100 g (lowest value recorded for a mixture of 50:50 wood particles and pine-cone flour). Buyuksari [29] studied the addition of pine-cone flour into particleboard that significantly decreased formaldehyde emissions. This ranged from 1.99 to 2.48 mg/100 g for various panel types (lowest value was recorded for a mixture of 50:50 wood particles and pine-cone flour).

Ayrilmis [30] analyzed the influence of pine-cone flour on the formaldehyde emissions of MDF and showed that the level of formaldehyde was 2.6% to 55.3% lower when the wood fibers were mixed with pine-cone flour. The decrease in the formaldehyde emission of panels containing the pine-cone particles was attributed to a high amount of phenolic extractives of the stone pine cones. These phenolic extractives are also components of the larch bark, so the same trend of reducing the formaldehyde content of the boards was observed. 

Salem [31] published an evaluation of formaldehyde content (FC) from different types of wood-based materials according to the perforator method (EN 120:2011). The highest FC amount was observed for the PB with a thickness of 25 mm, and reached 11.57 mg/100g oven dry (o.d.), which was above the E1 classification. MDF values ranged from 6.78 to 7.05 mg/100 g o.d. for the corrected perforator values, for a wide spectrum of thicknesses (3–22 mm). 

Pásztory [32] studied the formaldehyde absorption–desorption of poplar bark (*Populus × euramericana cv. Pannónia*) and found that, in a formaldehyde-polluted environment, bark samples could adsorb detectable formaldehyde amounts from contaminated air.

Weigl [33] reported about woodborne formaldehyde and the influence of species, wood grade, and cambial age on the amount of formaldehyde content. The lowest FC value was recorded for juvenile beech (0.15 mg/100 g), and the highest value was documented for mature pine wood (0.70 mg/100 g).

The aim of this study was to find to what extent larch bark acts as a formaldehyde scavenger in the case of 10 mm decorative larch-bark panels bonded with UF, polyvinyl acetate (PVAc), a mixture of 70% UF + 30% PVAc, polyurethane (PUR), tannin-based adhesive, and self-agglomeration. This study follows our previous research [34,35,36], where decorative larch-bark panels with different resins were tested for mechanical and physical properties. On the basis of this research, larch-bark panels that were bonded with different resins met the EN standards for elasticity modulus, bending strength, thickness swelling, and water uptake after 24 h.

## 2. Materials and Methods 

The analyzed tree bark for the manufacture of decorative boards in the present study was collected in a sawmill in Unternberg, Federal State of Salzburg, Austria, specialized in larch processing. For this reason, the raw material was not contaminated with other species. The bark was dried by means of a vacuum kiln dryer (Brunner–Hildebrand High VAC-S, HV-S1) from 100% to 9% moisture content. Drying temperature was 60 °C at a pressure of 200 to 250 mbar. The bark was subsequently crushed in a 4-spindle shredder (RS40) at the Untha Co. in Kuchl, Austria, and repeatedly screened in order to obtain 1.5 to 10 mm particles. For the manufacturing of the larch-bark-based panels, UF resin (10F102 MetaDynea Austria GmbH, Krems, Austria), with 66% solid content, pH 8.3–9 and viscosity 60–90 mPa.s, and tannin-based adhesive were used. Additionally, PVAc Kleiberit 303.3 (Klebchemie M.G. Becker GmbH and Co., Weingarten, Germany), with pH level and viscosity 3, and 12,000 ± 2000 mPa.s and PUR Kleiberit 501, 1.13 g/cm^3^ density and 8000 mPa.s viscosity were also chosen for the production of decorative panels.

The tannin-based adhesive was prepared with an extract powder from mimosa tannin (*Acacia mearnsii*) from Phenotan, Tanac SA, Brazil; hexa-methylenetetramine (hexamine) from Merck Schuchardt OHG, Germany (C99%); and sodium hydroxide solution (C32%) from Carl Roth GmbH and Co., Austria. We then stirred 50% tannin extract powder and 50% water with a mechanical mixer at a speed between 700 and 1500 rpm, and added 10% of hexamine to adjust the pH value of the mixed solution to 9 with a sodium hydroxide solution [11].

Boards of 10 mm thickness and with 0.6 g/cm^3^ density were produced with 10% resin content of 6 different adhesive systems, using 2.5–4 mm (fine) and 4–11 mm (coarse) larch-bark particle sizes (Table 1). Press temperature was 180 °C for the UF and tannin-based bonded boards, and 80 °C for the boards with other adhesive formulations. Moisture content (m.c.) was measured for each type of board (Table 1). 

Each board was cut into 2.5 × 2.5 mm samples after cooling. Samples were placed in airtight bags and delivered to the Kaindl Company, Wals, Salzburg, Austria, where formaldehyde content was measured. 

EN 120:2011 [37] was used to determine the formaldehyde content of the larch-bark panels. This method is suitable for nonlaminated and uncoated wood-based panels [38]. 

Small specimens (110 g of 25 × 25 mm) were extracted by means of boiling toluene, and then transferred into distilled or demineralized water. In the case of boards with low formaldehyde content, the mass of test samples could be extended up to 200 g, according to EN ISO 12460:5:2015 [39]. Formaldehyde emission was sampled through perforation in water and photometrically determined with the acethylacetone method. The perforator value depends on the moisture content of the specimens [32], which was determined according to ISO16999 (2003). The perforator values were corrected for boards conditioned to a moisture content of 6.5%. When the PB moisture content ranged between 3% and 10%, the EN 120 test value, if multiplied by a factor F, was calculated according to Equation (1) [31]:

F = −0.133 mc + 1.86.(1)

This method requires simple equipment and has a running time of 3 h, so is used on a large scale for production control in wood-based panels industry in Europe and China [32].

## 3. Results and Discussion

**Figure 1:**
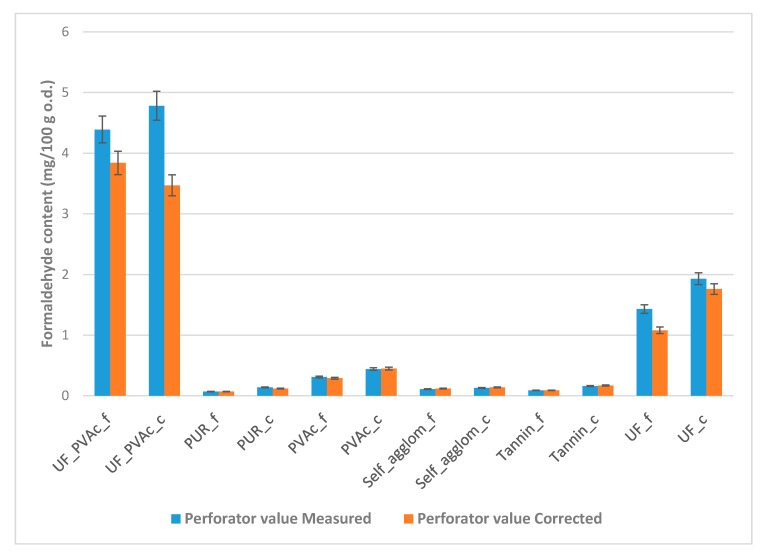
Free formaldehyde content for both measured and corrected perforator values (EN120:2011) of samples of larch-bark bonded decorative boards with six types of adhesives.

**Figure 2:**
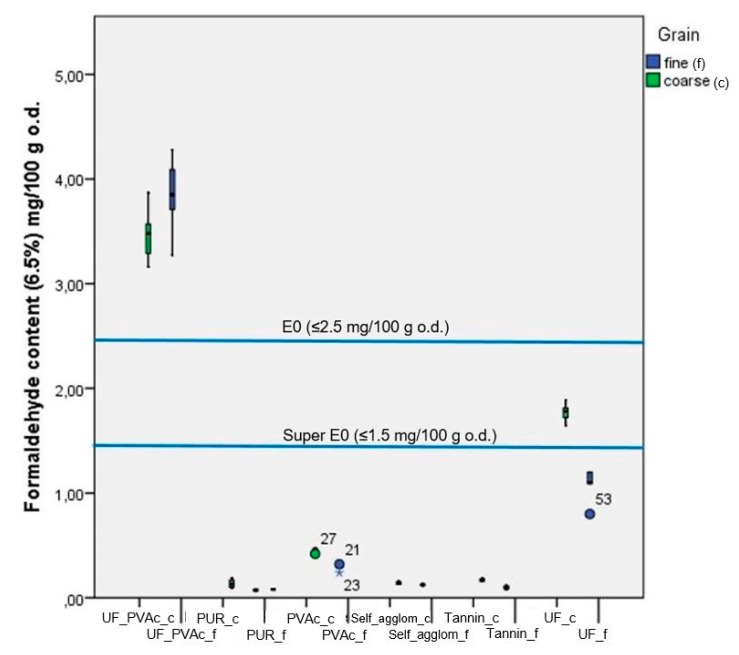
Free formaldehyde content measured according to EN 120:2011 of 10 mm larch-bark boards bonded with six types of adhesives.

The corrected values of free formaldehyde content varied depending on the type of adhesive formulation for the board (Figure 1). The lowest amounts were measured for the board with a fine fraction of larch bark bonded with PUR and tannin-based adhesive (0.07 and 0.09, respectively). Five types of panels registered values under super-E0 (≤1.5 mg/100 g): PUR_f, Tannin_f, PUR_c, Self_agglom_f and Self_agglom_c. At the boundary between super-E0 ((≤1.5 mg/100 g) and E0 (≤2.5 mg/100 g) was the board based on larch-bark particles (4–11 mm) glued with UF. The E0 class was reached by the PVAc (fine and coarse fraction) and UF (fine fraction) panels. When the larch-bark particles (both fractions) were glued with the mixture of UF (70%) and PVAc (30%), UF_PVAc_f and UF_PVAc_c, formaldehyde content had unexpectedly higher values (<4 mg/100 g) than all other tested samples, but was still about half the value of the standard wood-based panels that should have the E1 emission class (≤8 mg/100 g; Figure 2).

**Figure 3:**
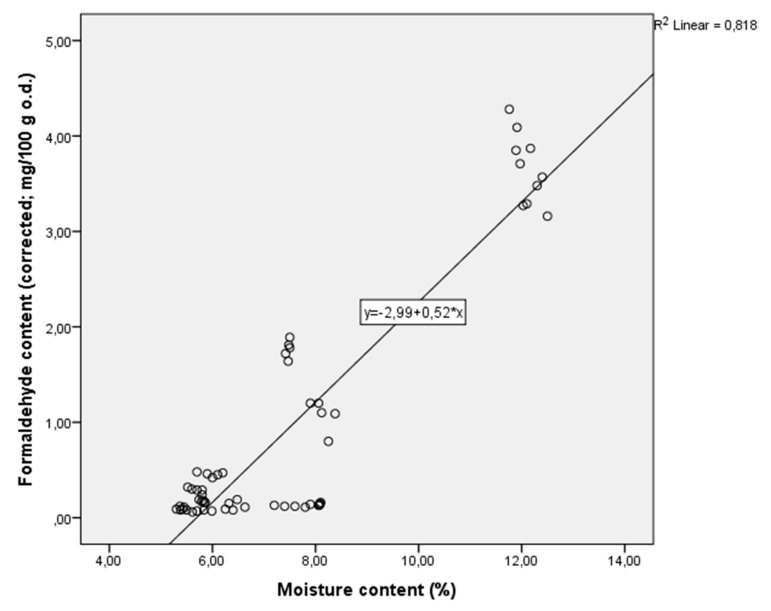
Normal P-P plot of regression-standardized residual for corrected formaldehyde content of larch-bark samples bonded with six types of adhesives.

A multiple regression model (Figure 3) was calculated for all boards to observe the effect of board-moisture content and larch-bark particle fraction on formaldehyde content as measured by EN 120:2011. Moisture content was significantly correlated to the free formaldehyde content, with adjusted coefficient of determination R^2^ = 0.812 (Y = 0.5X − 2.99). Board-moisture content had a highly significant effect (*p* = 0.000), while the effect of particle fraction on formaldehyde content (*p* = 0.806) had no statistical impact. The influence of moisture content on FC was predominant for boards bonded with formaldehyde-based resin (UF and UF + PVAc).

The boards that reached super-E0 emission class were bonded with tannin and PUR. Self_agglom_f and Self_agglom_c (boards produced without adhesives) were included in the same classification, but in this case, a small amount of water (difference to 100% from the solid content of the UF adhesive) was added. The lignin from bark acted as natural glue due to its phenolic nature [40,41] and due to the self-bonding mechanisms of bark [42]. Tannin had the formaldehyde-scavenger effect for boards bonded with UF (e.g., MDF) [20]. In the case of the fine fraction of larch bark, tannin-bonded panels had a lower formaldehyde content compared with boards bound with other types of glue, and its natural lignin was “activated” with water.

The nonparametric Kolmogorov–Smirnov test for FC values (measured and calculated) revealed that both test distributions were normal (*p* = 0.000).

In order to compare the achieved values with nonbark-wood-based panels, we selected from the literature objective values of free formaldehyde content in decorative boards that were not distorted by local laboratory conditions, varying technical parameters of the used materials, or other factors. There are many studies [43,44,45,46,47,48,49] that showed that free-formaldehyde-content values in nonbark-wood-based panels were far higher than those in the larch-bark panels in this research. 

Many countries established formaldehyde-emission requirements and testing methods of formaldehyde emission from wood-based panels. A very successful comparison of the relationship between different methods and standard limits was made by Carvalho et al. [50], who obtained values by correlation of many observations and adapted certain values from Harmon [51]. In Europe, harmonized European standard EN 13986 classifies formaldehyde emission into E1 and E2, but it was recently agreed to only produce the E1 class, abandoning the production of E2 class panels. That corresponds to a perforator value below 8 mg/100 g oven-dry wood for PB (thickness > 8 mm). Driven by IKEA, an equivalent class with half E1 formaldehyde-emission limits was introduced: the so-called E0.5 that corresponds to a perforator value below 4 mg/100 g [52,53]. In a study conducted by Salem et al. [49], the authors recalculated the corresponding values from Japanese standards, which should be below 4.5 mg/100 g (and for special boards, a stricter limit of 2.7 mg/100 g), and U.S. standards, whose values would be at the level below 11.3 mg/100 g (and for special boards, a stricter limit of 5.6 mg/100 g). The summary results in Figure 1 show the significant benefit of raw larch-bark material used to reduce free formaldehyde in decorative panels in our research compared with standard global limits. Fortunately, larch bark performed excellently in the perforator test, the most efficient method used in the industry for PB and MDF to give rapid feedback about the formaldehyde content of the composite. It would be interesting to study to what extent other types of tree bark are suitable for the manufacture of boards with similar lower formaldehyde content.

## 4. Conclusions

Free formaldehyde content (according to EN 120:2011) was determined for larch-bark panels with the same thickness (10 mm) and density (0.6 g/cm^3^) for a fine and coarse grain and different adhesives. Following the results, the following findings can be stated:

- Larch bark had significant influence on the characteristics of free formaldehyde content in decorative panels. It was found that the majority of the panel samples were included in the E0 category (≤2.5 mg/100 g o.d.). The values of free formaldehyde content varied depending on the type of adhesive formulation for the board. It can be considered a significant contribution in terms of the environment.
- In addition, the research identified that 75% of the test specimens bonded with tannin and PUR adhesives reached super-E0 classification (≤1.5 mg/100 g o.d.). These new classes (E0 and super-E0) of low formaldehyde emitting panels were initiated by the Japanese standards (JIS), and established by the European Panel Federation (EPF).
- The multiple-regression model, calculated for all boards to observe the effect of board-moisture content and larch-bark particle fraction on formaldehyde content, indicated that moisture content is significantly correlated with free formaldehyde content, with adjusted coefficient of determination R^2^ = 0.812 (Y = 0.5X − 2.99). Board-moisture content had a highly significant effect (*p* = 0.000), while the effect of particle fraction on formaldehyde content (*p* = 0.806) had no statistical impact.
- The bark of the selected wood species had a positive impact on free formaldehyde reduction, providing grounds for further research.

## Figures and Tables

**Table 1:** Experiment design with factor larch-bark particle size (two replications for each decorative board). Note: PUR, polyurethane; PVAc, polyvinyl acetate; UF, urea-formaldehyde.

Board	Glue	Moisture Content (M. C.) %	Particle Size mm
UF_PVAc_f	70% UF + 30% PVAc	7.65	2.5–4
UF_PVAc_ce	70% UF + 30% PVAc	8.42	4–11
PUR_f	PUR	5.97	2.5–4
PUR_c	PUR	4.21	4–11
PVAc_f	PVAc	4.43	2.5–4
PVAc_c	PVAc	4.83	4–11
Self_agglom_f	Water	9.17	2.5–4
Self_agglom_c	Water	7.73	4–11
Tannin_f	Tannin	8.45	2.5–4
Tannin_c	Tannin	8.45	4–11
UF_f	UF	8.45	2.5–4
UF_c	UF	8.45	4–11
